# Email-Based Recruitment Into the Health eHeart Study: Cohort Analysis of Invited Eligible Patients

**DOI:** 10.2196/51238

**Published:** 2023-12-22

**Authors:** Madelena Y Ng, Jeffrey E Olgin, Gregory M Marcus, Courtney R Lyles, Mark J Pletcher

**Affiliations:** 1 School of Public Health University of California Berkeley, CA United States; 2 Department of Medicine (Biomedical Informatics) Stanford University Stanford, CA United States; 3 Department of Medicine University of California San Francisco, CA United States; 4 Department of Public Health Sciences University of California Davis, CA United States; 5 Department of Epidemiology and Biostatistics University of California San Francisco, CA United States

**Keywords:** digital health study, recruitment, research participants, campaign evaluation, email, advertisement, enrollment, registration, consent, participation, engagement, eHealth

## Abstract

**Background:**

Web- or app-based digital health studies allow for more efficient collection of health data for research. However, remote recruitment into digital health studies can enroll nonrepresentative study samples, hindering the robustness and generalizability of findings. Through the comprehensive evaluation of an email-based campaign on recruitment into the Health eHeart Study, we aim to uncover key sociodemographic and clinical factors that contribute to enrollment.

**Objective:**

This study sought to understand the factors related to participation, specifically regarding enrollment, in the Health eHeart Study as a result of a large-scale remote email recruitment campaign.

**Methods:**

We conducted a cohort analysis on all invited University of California, San Francisco (UCSF) patients to identify sociodemographic and clinical predictors of enrollment into the Health eHeart Study. The primary outcome was enrollment, defined by account registration and consent into the Health eHeart Study. The email recruitment campaign was carried out from August 2015 to February 2016, with electronic health record data extracted between September 2019 and December 2019.

**Results:**

The email recruitment campaign delivered at least 1 email invitation to 93.5% (193,606/206,983) of all invited patients and yielded a 3.6% (7012/193,606) registration rate among contacted patients and an 84.1% (5899/7012) consent rate among registered patients. Adjusted multivariate logistic regression models analyzed independent sociodemographic and clinical predictors of (1) registration among contacted participants and (2) consent among registered participants. Odds of registration were higher among patients who are older, women, non-Hispanic White, active patients with commercial insurance or Medicare, with a higher comorbidity burden, with congestive heart failure, and randomized to receive up to 2 recruitment emails. The odds of registration were lower among those with medical conditions such as dementia, chronic pulmonary disease, moderate or severe liver disease, paraplegia or hemiplegia, renal disease, or cancer. Odds of subsequent consent after initial registration were different, with an inverse trend of being lower among patients who are older and women. The odds of consent were also lower among those with peripheral vascular disease. However, the odds of consent remained higher among patients who were non-Hispanic White and those with commercial insurance.

**Conclusions:**

This study provides important insights into the potential returns on participant enrollment when digital health study teams invest resources in using email for recruitment. The findings show that participant enrollment was driven more strongly by sociodemographic factors than clinical factors. Overall, email is an extremely efficient means of recruiting participants from a large list into the Health eHeart Study. Despite some improvements in representation, the formulation of truly diverse studies will require additional resources and strategies to overcome persistent participation barriers.

## Introduction

Research participants are increasingly approached through digital or remote means to contribute their health data for biomedical discovery and innovation. Digital research studies delivered through a web portal (web-based) or smartphone app (app-based) aim to harness the capabilities and ubiquity of the internet, smartphones, and sensor devices to improve recruitment, engagement, and data collection over time [1-5]. Digital health studies can eliminate structural barriers to participation, provide more accessible support and feedback to participants, and allow for the efficient collection of frequent and real-time health data [2-5].

Recruitment of a representative study population is critical for achieving the goals of precision medicine and ensuring the generalizability of findings [6,7]. However, persistent recruitment challenges remain with digital health studies. In addition to being similarly vulnerable to selection biases (eg, altruistic volunteers) and participation attrition as traditional clinical research studies, they must also contend with additional biases and inequities arising from the digital environment [2,3,8]. Recruited participants in digital health studies also tend to be those who are non-Hispanic White, have higher income, and educational levels [3,9], which are groups that are already better represented in conventional health research [10,11]. Given the value of remote recruitment through the internet and the potential pitfalls, enriching our understanding of key selection biases in this context is crucially important.

We conducted a broad-based digital recruitment campaign within our health system, sending unsolicited email invitations to over 200,000 patients between August 2015 and February 2016 inviting them to join the Health eHeart Study (a cardiovascular-focused “eCohort” with registration, consent, and ongoing data collection occurring entirely on the internet). Random subsets of the sampling frame received emails on different days, at different times, and with different subject lines to maximize variation in the recruitment outreach strategy. To study the success of our campaign in recruiting different segments of our target population, we evaluated our invited eligible patient cohort at the earliest point of research participation—enrollment—defined by registration and consent into the Health eHeart Study. We obtained electronic health record data from the entire sampling frame and used these data to analyze sociodemographic and clinical characteristics of the patients that predicted Health eHeart Study enrollment and analyzed barriers at each step in the enrollment process, with the goal of learning how email-based recruitment can be used optimally to enroll representative study populations for digital health studies.

## Methods

### Study Design and Population

Eligible patients from the University of California, San Francisco (UCSF) Medical Center were invited to join the Health eHeart Study [12] via an email recruitment campaign carried out from August 2015 to February 2016. The Health eHeart Study is a worldwide digital cardiovascular health–focused electronic cohort (eCohort) coordinated at the UCSF. Participation in the Health eHeart Study is open to adults (age ≥18 years) who understand English and have an active email. Health eHeart participants are recruited through a variety of modes, including clinic visits, word-of-mouth, lay press, social media, promotional events, and email. After web-based registration (name, date of birth, email, and password) and consent, participants are prompted to complete web-based surveys about their basic and social demographics, medical history, family history, activity and well-being, habits and lifestyle, mental health, diet and nutrition, and technology use. Participants also have the option to “connect” mobile health devices and apps to contribute additional data to the study. The Health eHeart Study’s design and procedures (eg, user interfaces, incentives, technical support, and reminder schedule) were not preferentially altered for the email recruitment campaign or subgroups of the invited patients.

### Ethical Considerations

The UCSF institutional review board approved both the Health eHeart Study and the analysis of this digital recruitment campaign (#15-18180). Ethics approval covers secondary data analyses without additional consent in accordance with institutional guidelines. Protective measures (eg, encryption) were carried out to safeguard all study data.

### Email Recruitment Campaign Plan

Patients are defined as those who have a patient record within the UCSF electronic health record (EHR) system. Living UCSF patients who are 18 years of age or older, with a documented email address within the EHR, and with English recorded as their preferred language were sent an email invitation (Multimedia Appendix 1). The email invitation included a short description about the study mission and a “call-to-action” button for patients to register. The button launched a browser with a patient-specific URL including a linkage identifier that enabled the Health eHeart Study to link their Health eHeart account to the specific UCSF patient receiving the email. Emails were designed and scheduled through Mailchimp (Intuit), an email marketing platform that also provides recipient delivery and engagement metrics. The email campaign was segmented into 15 initial “waves” of recruitment (Multimedia Appendix 2). Patients for each wave were randomly selected. Different waves also received varying subject line messaging and delivery days and times to maximize variation in the recruitment outreach strategy. Patients from waves 1 to 14 were sent 1 follow-up email invitation if they remained unregistered (excluding unsubscribes and hard bounces) for at least 2 weeks after the initial invitation.

### Study Setting

We conducted a cohort analysis of all invited UCSF patients to identify predictors of enrollment. Data were extracted from the UCSF EHR system (Epic Systems Corporation) between September 2019 and December 2019, for sociodemographic, clinical diagnoses, health care use, and insurance coverage information at the time of the patient’s email invitation date. We limited the analysis to contacted patients, defined as those sent an email via Mailchimp that was not rejected by the patient’s email server.

### Outcomes

The primary outcome of interest was enrollment, defined by account registration and consent into the Health eHeart Study, among contacted patients. In the Health eHeart Study, enrollment occurs in 2 crucial steps: registration and consent. We also looked at these 2 steps separately. We defined registered patient participants as those who set up a Health eHeart Study account with a name, date of birth, email, and password. We defined consented patient participants as registered patients who indicated their willingness to participate, after being shown the consent form, by clicking on an “I want to participate” button.

### Independent Variables and Covariates

Patient-level sociodemographic variables from the EHR include (1) age (<30, 30-39, 40-49, 50-59, 60-69, 70-79, ≥80 years); (2) sex (male and female); (3) race or ethnicity (non-Hispanic White, non-Hispanic Black, non-Hispanic Asian or Asian and Pacific Islander [API], Hispanic or Latino, other, multiracial, and unknown or declined to state); (4) insurance (commercial, Medicaid, Medicare, other, and unknown or declined to state); and (5) marital status (married or partnered, not married or partnered, and unknown or declined to state). Insurance status serves as a proxy for individual socioeconomic status (SES) since income levels cannot be ascertained from the EHR. Insurance statuses of Medicaid or unknown or declined to state were used as indicators of lower SES.

We derived the following patient-level clinical variables using EHR data extracted from UCSF’s clinical data warehouse: (1) Charlson Comorbidity Index (CCI) score (0, 1, 2, and 3+); (2) diagnosis of medical condition (myocardial infarction, congestive heart failure, peripheral vascular disease, cerebrovascular disease, dementia, chronic pulmonary disease, rheumatologic disease, peptic ulcer disease, diabetes, liver disease (mild, moderate, or severe), paraplegia or hemiplegia, cancer (any type or metastatic solid tumor), renal disease, HIV/AIDS); and (3) UCSF recent patient status (inactive and active). Patients are defined as “active” if they had 1 or more health care encounters at UCSF 6 months prior to the initial email invitation. The CCI score is a validated standardized measure of overall comorbidity burden and generates a weighted index based on the relative risks of 1-year mortality or “the number and seriousness” of 17 comorbid diseases [13,14]. CCI scores were calculated at the time of the initial contact email and therefore represent each patient’s comorbidity status at the time of recruitment.

For recruitment-related variables, we also adjusted for the number of recruitment emails received (1 vs 2). Patients in wave 15 were only delivered 1 (initial) email, while those in waves 1 to 14 had the potential to be delivered up to 2 (initial and follow-up) email invitations.

### Statistical Analysis

Descriptive statistics were performed to characterize sociodemographic and clinical distributions for (1) all contacted patients, (2) registration status among contacted patients, and (3) consent status among registered patients. Patient characteristics were compared between (1) registered versus did not register and (2) consented versus did not consent using bivariate analysis. Categorical variables are presented as frequencies (percentages) and compared using chi-square tests. Continuous variables with skewed distributions are presented as medians (IQR). We used logistic regression to simultaneously adjust for all patient- and email campaign–related variables. We first performed a univariate (unadjusted) analysis for each variable (Multimedia Appendix 3), then performed a multivariate (adjusted) analysis that included all variables. The results are summarized as unadjusted odds ratios or adjusted odds ratios (aORs), respectively, with 95% CIs. All statistical analyses were conducted using STATA (version 16.0; StataCorp), and *P* values <.05 were considered statistically significant.

## Results

### Recruitment

**Figure 1:**
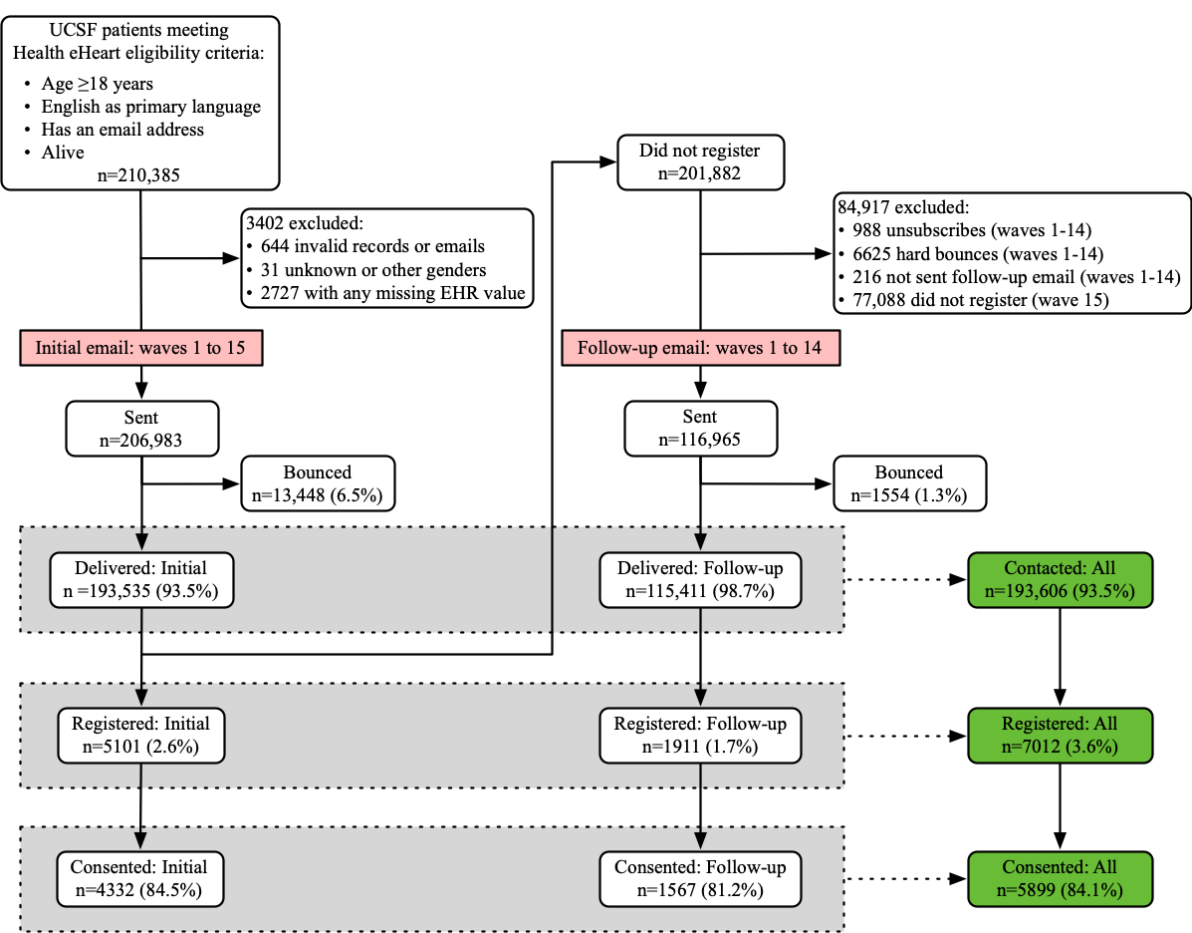
Flowchart of Health eHeart Study’s email recruitment campaign delivered to UCSF patients who satisfied the study eligibility criteria. EHR: electronic health record; UCSF: University of California, San Francisco.

Figure 1 shows the flow of recruited patients from invitation to registration and web-based informed consent. An EHR data search generated a recruitment list of 210,385 eligible patients. Due to invalid or incomplete records, 3402 patients were excluded from recruitment or analysis. An initial recruitment email was sent in 15 waves to a total of 206,983 patients, where approximately 6.5% (n=13,448) returned a bounced email, resulting in an initial contact rate of 93.5% (n=193,535). The primary barrier to enrollment was registration (Figure 1). Among those initially contacted, only 2.6% (5101/193,535) registered, of which 84.5% (4332/5101) consented.

A follow-up recruitment email was sent to a total of 116,965 patients from waves 1 to 14, who were initially contacted but did not register or whose initial email bounced. Approximately 1.3% (n=1554) returned a bounced email, resulting in a follow-up contact rate of 98.7% (n=115,411). Among those recontacted, 1.7% (1911/115,411) registered, of which 81.2% (1567/1911) consented.

Overall, the email recruitment campaign delivered at least 1 email invitation to 93.5% (193,606/206,983) of all recruited patients, which yielded a 3.6% (7012/193,606) registration rate among contacted patients and an 84.1% (5899/7012) consent rate among registered patients. The overall enrollment rate for registration and consent was 3.6% (7012/193,606) and 3% (5899/193,606) among ever-contacted participants, respectively.

### Patient Characteristics

Patient characteristics for all contacted patients, by their registration and consent status, are reported in Table 1. In terms of sociodemographic characteristics, the median (IQR) age of 193,606 contacted patients was 48.5 (36.0-62.6) years, with the majority being non-Hispanic White (n=115,418, 59.6%), women (n=113,455, 58.6%) with commercial insurance (n=107,972, 55.8%), married or partnered (n=100,071, 51.7%). Approximately half (n=96,516, 49.9%) were considered active patients of UCSF and 62.1% (120,149/193,606) were part of a wave with scheduled follow-up emails. In terms of clinical characteristics, while the majority (124,861/193,606, 64.5%) of contacted patients had a CCI score of 0 (ie, no comorbidity burden or severity), the most reported medical conditions among contacted patients were cancer (28,677/193,606, 14.8%) and chronic pulmonary disease (18,771/193,606, 9.7%). Table 1 also shows how sociodemographic and clinical characteristics significantly differed by registration status among all contacted patients and consent status among registered patients.

**Table 1:** Descriptive statistics of all contacted patients by registration and consent status.

Characteristics			All recruited		Registration status								Consent status						
			Contacted (N=193,606), n (%)		Did not register (n=186,594), n (%)		Registered (n=7012), n (%)		*P* value			Did not consent (n=1113), n (%)			Consented (n=5899), n (%)			*P* value	
**Age group (years)**										<.001									<.001
	<30	24,111 (12.5)		23,732 (12.7)		379 (5.4)					36 (3.2)			343 (5.8)					
	30-39	40,488 (20.9)		39,783 (21.3)		705 (10.1)					93 (8.4)			612 (10.4)					
	40-49	36,937 (19.1)		35,923 (19.3)		1014 (14.5)					145 (13)			869 (14.7)					
	50-59	34,474 (17.8)		33,070 (17.7)		1404 (20)					246 (22.1)			1158 (19.6)					
	60-69	33,740 (17.4)		31,783 (17)		1957 (27.9)					327 (29.4)			1630 (27.6)					
	70-79	18,213 (9.4)		16,937 (9.1)		1276 (18.2)					212 (19)			1064 (18)					
	≥80	5643 (2.9)		5366 (2.9)		277 (4)					54 (4.9)			223 (3.8)					
**Sex**										<.001									<.001
	Male	80,151 (41.4)		77,488 (41.5)		2663 (38)					355 (31.9)			2308 (39.1)					
	Female	113,455 (58.6)		109,106 (58.5)		4349 (62)					758 (68.1)			3591 (60.9)					
**Race or ethnicity**										<.001									<.001
	Asian or API^a^, non-Hispanic	23,578 (12.2)		23,105 (12.4)		473 (6.7)					97 (8.7)			376 (6.4)					
	Black, non-Hispanic	7703 (4)		7550 (4)		153 (2.2)					38 (3.4)			115 (1.9)					
	Hispanic or Latino	13,070 (6.8)		12,678 (6.8)		392 (5.6)					89 (8)			303 (5.1)					
	White, non-Hispanic	115,418 (59.6)		110,300 (59.1)		5118 (73)					727 (65.3)			4391 (74.4)					
	Multiracial	4056 (2.1)		3934 (2.1)		122 (1.7)					23 (2.1)			99 (1.7)					
	Other	11,598 (6)		11,299 (6.1)		299 (4.3)					62 (5.6)			237 (4)					
	Unknown or declined to state	18,183 (9.4)		17,728 (9.5)		455 (6.5)					77 (6.9)			378 (6.4)					
**Insurance**										<.001									<.001
	Commercial	107,972 (55.8)		104,359 (55.9)		3613 (51.5)					517 (46.5)			3096 (52.5)					
	Medicaid	13,706 (7.1)		13,378 (7.2)		328 (4.7)					71 (6.4)			257 (4.4)					
	Medicare	35,484 (18.3)		33,269 (17.8)		2215 (31.6)					388 (34.9)			1827 (31)					
	Other	9298 (4.8)		9053 (4.9)		245 (3.5)					39 (3.5)			206 (3.5)					
	Unknown or declined to state	27,146 (14)		26,535 (14.2)		611 (8.7)					98 (8.8)			513 (8.7)					
**Marital status**										<.001									.38
	Married or partnered	100,071 (51.7)		96,028 (51.5)		4043 (57.7)					622 (55.9)			3421 (58)					
	Not married or partnered	84,022 (43.4)		81,338 (43.6)		2684 (38.3)					441 (39.6)			2243 (38)					
	Unknown or declined to state	9513 (4.9)		9228 (4.9)		285 (4.1)					50 (4.5)			235 (4)					
**Wave with follow-up**										<.001									.77
	No (wave 15)	73,457 (37.9)		71,681 (38.4)		1776 (25.3)					278 (25)			1498 (25.4)					
	Yes (waves 1-14)	120,149 (62.1)		114,913 (61.6)		5236 (74.7)					835 (75)			4401 (74.6)					
**Patient status**										<.001									.07
	Inactive patients	97,090 (50.1)		94,570 (50.7)		2520 (35.9)					373 (33.5)			2147 (36.4)					
	Active patients	96,516 (49.9)		92,024 (49.3)		4492 (64.1)					740 (66.5)			3752 (63.6)					
**CCI^b^ score**										<.001									.32
	0	124,861 (64.5)		120,823 (64.8)		4038 (57.6)					620 (55.7)			3418 (57.9)					
	1	23,339 (12.1)		22,378 (12)		961 (13.7)					148 (13.3)			813 (13.8)					
	2	20,829 (10.8)		19,845 (10.6)		984 (14)					166 (14.9)			818 (13.9)					
	3+	24,577 (12.7)		23,548 (12.6)		1029 (14.7)					179 (16.1)			850 (14.4)					
**Medical conditions**																			
	Myocardial infarction	2184 (1.1)		2047 (1.1)		137 (2)		<.001			23 (2.1)			114 (1.9)			.77		
	Congestive heart failure	4290 (2.2)		4023 (2.2)		267 (3.8)		<.001			41 (3.7)			226 (3.8)			.81		
	Peripheral vascular disease	3541 (1.8)		3334 (1.8)		207 (3)		<.001			47 (4.2)			160 (2.7)			.01		
	Cerebrovascular disease	6339 (3.3)		6020 (3.2)		319 (4.5)		<.001			64 (5.8)			255 (4.3)			.04		
	Dementia	1204 (0.6)		1168 (0.6)		36 (0.5)		.24			4 (0.4)			32 (0.5)			.43		
	Chronic pulmonary disease	18,771 (9.7)		18,036 (9.7)		735 (10.5)		.02			123 (11.1)			612 (10.4)			.50		
	Rheumatic disease	3134 (1.6)		2952 (1.6)		182 (2.6)		<.001			54 (4.9)			128 (2.2)			.98		
	Peptic ulcer disease	1604 (0.8)		1543 (0.8)		61 (0.9)		.70			10 (0.9)			51 (0.9)			.91		
	Diabetes	12,407 (6.4)		11,844 (6.3)		563 (8)		<.001			105 (9.4)			458 (7.8)			.06		
	Mild liver disease	8213 (4.2)		7900 (4.2)		313 (4.5)		.35			62 (5.6)			251 (4.3)			.05		
	Moderate or severe liver disease	1662 (0.9)		1615 (0.9)		47 (0.7)		.08			13 (1.2)			34 (0.6)			.03		
	Paraplegia or hemiplegia	975 (0.5)		952 (0.5)		23 (0.3)		.03			2 (0.2)			21 (0.4)			.35		
	Renal disease	8100 (4.2)		7787 (4.2)		313 (4.5)		.23			55 (4.9)			258 (4.4)			.40		
	Cancer	28,677 (14.8)		27,422 (14.7)		1255 (17.9)		<.001			207 (18.6)			1048 (17.8)			.51		
	Metastatic solid tumor	9743 (5)		9345 (5)		398 (5.7)		.01			65 (5.8)			333 (5.6)			.80		
	HIV/AIDS	1812 (0.9)		1748 (0.9)		64 (0.9)		.84			8 (0.7)			56 (0.9)			.46		

^a^API: Asian and Pacific Islander.

^b^CCI: Charlson Comorbidity Index.

### Characteristics Associated With Health eHeart Study Registration

**Figure 2:**
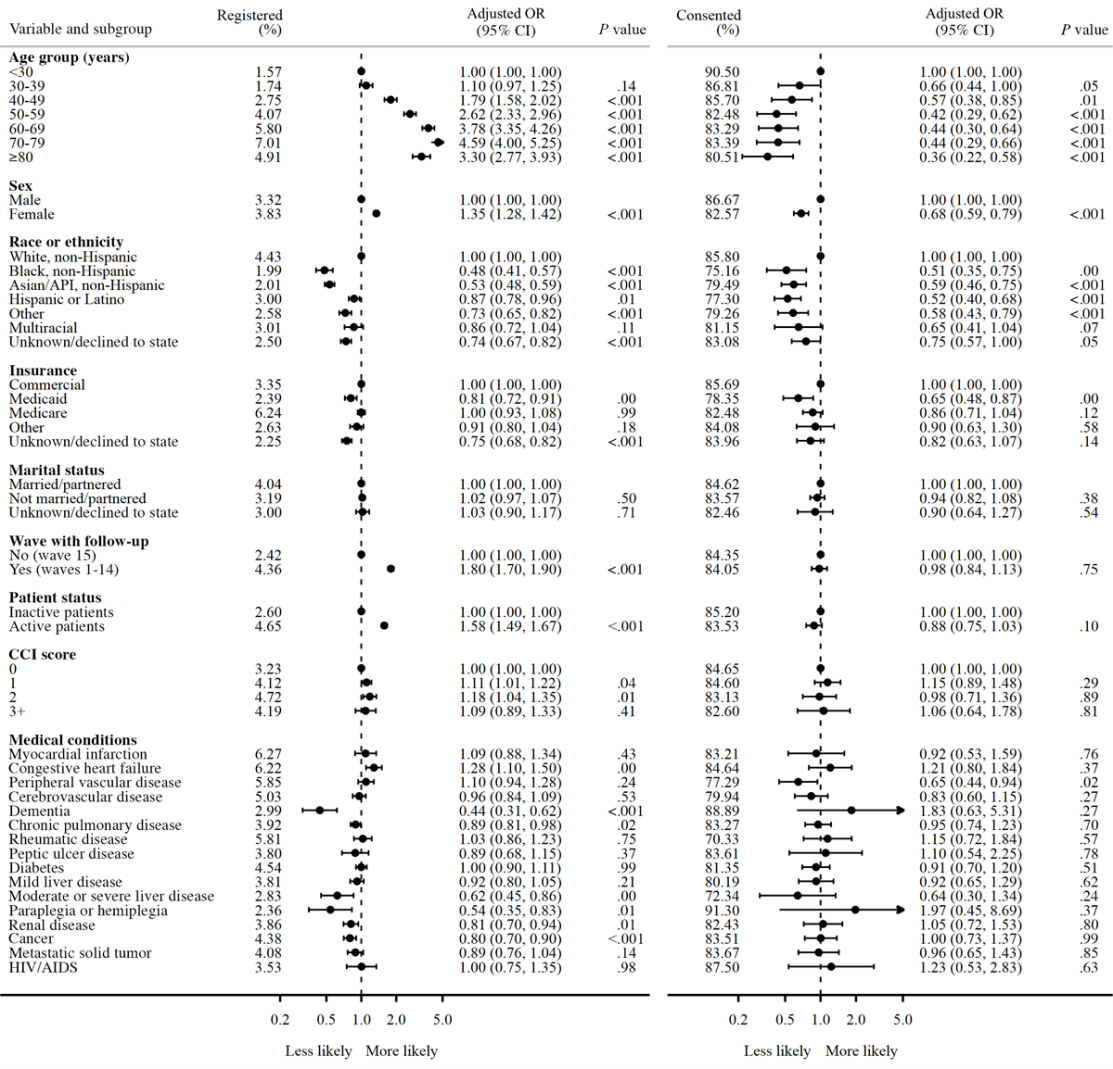
Adjusted odds of registration among contacted patients (left) and consent among registered patients (right).

Figure 2 presents the results from 2 adjusted multivariate logistic regression models. The first model analyzes independent predictors of registration among contacted participants; the second model analyzes predictors of consent among registered participants. All variables were significant in the first registration logistic model, controlling for all other variables in the equation, except for marital status and certain medical conditions.

Higher age groups were associated with greater odds of registration; compared to those <30 years, the odds of registration was significantly higher for every decade increase in age from 40-49 (aOR 1.79, 95% CI 1.58-2.02), 50-59 (aOR 2.62, 95% CI 2.33-2.96), and 60-69 (aOR 3.78, 95% CI 3.35-4.26) to 70-79 (aOR 4.59, 95% CI 4.00-5.25), before waning slightly for those ≥80 years (aOR 3.30, 95% CI 2.77-3.93). Relative to men, women (aOR 1.35, 95% CI 1.28-1.42) had greater odds of registration. Compared to non-Hispanic White patients, the odds of registration were lower among patients who were non-Hispanic Black (aOR 0.48, 95% CI 0.41-0.57), non-Hispanic Asian or API (aOR 0.53, 95% CI 0.48-0.59), Hispanic or Latino (aOR 0.87, 95% CI 0.78-0.96), other (aOR 0.73, 95% CI 0.65-0.82), or unknown or declined to state (aOR 0.74, 95% CI 0.67-0.82) race or ethnicity. Relative to those with commercial insurance, those with Medicaid (aOR 0.81, 95% CI 0.72-0.91) or unknown or declined to state (aOR 0.75, 95% CI 0.68-0.82) insurance had lower odds of registration.

Those from waves 1 to 14 (aOR 1.80, 95% CI 1.70-1.90), which included a follow-up email, had greater odds of registration than those from wave 15, where there was no follow-up email. Active UCSF patients (aOR 1.58, 95% CI 1.49-1.67) had greater odds of registration compared to inactive patients. Compared to those with a CCI score of 0, patients with some comorbidity were more likely to register (aOR 1.11, 95% CI 1.01-1.22 for a score of 1 or aOR 1.18, 95% CI 1.04-1.35 for a score of 2). A diagnosis of congestive heart failure (aOR 1.28, 95% CI 1.10-1.50) was independently associated with greater odds of registration, while a diagnosis of dementia (aOR 0.44, 95% CI 0.31-0.62), chronic pulmonary disease (aOR 0.89, 95% CI 0.81-0.98), moderate or severe liver disease (aOR 0.62, 95% CI 0.45-0.86), paraplegia or hemiplegia (aOR 0.54, 95% CI 0.35-0.83), renal disease (aOR 0.81, 95% CI 0.70-0.94), or any type of cancer (aOR 0.80, 95% CI 0.70-0.90) were associated with lower odds of registration after adjustment for overall comorbidity and age (both strongly associated with specific conditions).

### Characteristics Associated With Consent Among Registered Patients

Only sociodemographic variables (except for marital status) and 1 medical condition were significantly associated with consent among registered patients after adjusting for all other variables in the equation.

Higher age groups were associated with lower odds of consent; compared to those <30 years, the odds of consent significantly decreased in age groups from 30-39 (aOR 0.66, 95% CI 0.44-1.00), 40-49 (aOR 0.57, 95% CI 0.38-0.85), 50-59 (aOR 0.42, 95% CI 0.29-0.62), 60-69 (aOR 0.44, 95% CI 0.30-0.64), 70-79 (aOR 0.44, 95% CI 0.29-0.66) to ≥80 (aOR 0.36, 95% CI 0.22-0.58) years. Relative to men, women had (aOR 0.68, 95% CI 0.59-0.79) lower odds of consent. Compared to non-Hispanic White patients, the odds of consent were lower among patients who were non-Hispanic Black (aOR 0.51, 95% CI 0.35-0.75), non-Hispanic Asian or API (aOR 0.59, 95% CI 0.46-0.75), Hispanic or Latino (aOR 0.52, 95% CI 0.40-0.68), other (aOR 0.58, 95% CI 0.43-0.79), or unknown or declined to state (aOR 0.75, 95% CI 0.57-1.00) race or ethnicity. Relative to those with commercial insurance, those with Medicaid (aOR 0.65, 95% CI 0.48-0.87) had lower odds of consent. A diagnosis of peripheral vascular disease (aOR 0.65, 95% CI 0.44-0.94) was also associated with lower odds of consent.

The effect sizes for both models—registration and consent—are displayed alongside each other to show the scale of differences between them. Unadjusted models are included in Multimedia Appendix 3.

## Discussion

### Principal Findings

We set out to describe the sociodemographic and clinical characteristics that predict enrollment in the Health eHeart Study with an email-based recruitment campaign to patients in a health system. Initial engagement rates (registration among all contacted patients) were generally low (7012/193,606, 3.6%), with higher rates among patients who are older, women, non-Hispanic White, active patients with commercial insurance or Medicare, with a higher comorbidity burden, with congestive heart failure, and randomized to receive up to 2 recruitment emails. Patterns of subsequent consent after initial registration were somewhat different (with inverse trends by age and gender), but enrollment was strongly driven by initial engagement since most initially engaged patients end up consenting to join the study (5899/7012, 84.1%). Overall, enrollment was also driven more strongly by sociodemographic than clinical factors.

Email is an extremely efficient means of recruiting participants from a large list into the Health eHeart Study, a digital health study. The email recruitment campaign required relatively low operational effort from the research team and cost less than US $1000 to carry out through Mailchimp. The campaign led to 7012 patients registered, 5899 of whom consented, into the Health eHeart Study within 6 months. With access to a massive email list, the campaign was able to recruit a sufficient absolute number of total participants for most clinical trial needs. However, it also resulted in a lower proportion of patients who are racial or ethnic minorities and of lower SES. Despite email showing some advantages with recruitment, the underrepresentation of racial or ethnic minorities and those of lower SES was found to persist even at these early enrollment stages of a digital research study.

### Comparison With Prior Work

The extent to which older patients registered into the Health eHeart Study compared to their younger counterparts supports the notion that remote recruitment via email can be particularly effective in enrolling older people in the right context. This can be partially attributed to the study’s disease focus and institutional affiliation; heart health may inherently be more interesting to older patients seeking cardiovascular care at a research hospital compared to older people exploring a public forum. This finding contrasts with literature showing that those who enrolled in a research study through various digital channels tend to veer younger relative to the recruited population [15-18]. Historically, older adults are underrepresented in clinical trials, but this may be attributed to ageism and the associated biases and assumptions that hinder their recruitment [10,11,19]. One assumption is that older adults are more apprehensive toward technology; hence their underrepresentation may persist in digital health studies, but studies have shown that that barrier is smaller than once imagined [17,20]. The COVID-19 pandemic also accelerated older adults’ adoption of digital health technologies, furthering the need to update recruitment approaches to maximize their inclusion in digital research [19].

Female patients, to a lesser effect, were also more likely to participate in the Health eHeart Study compared to male patients. The underrepresentation of women in conventional studies has been partially attributed to structural factors, such as time demands and scheduling, study type (eg, randomized trial), financial incentives, seasonality, and clinical environment, among many other reasons [21-23]. In contrast, women were consistently reported to be more likely to participate in digital research studies [3,24], which can help bypass structural barriers to research participation. The findings from the email campaign further support the reversal in gender representation in digital research studies.

Consistent with existing trends [25,26], patients who identified as racial or ethnic minorities were noticeably less inclined to participate in the Health eHeart Study compared to non-Hispanic White patients at both the registration and consent steps. Even though racial or ethnic minorities have indicated to be as willing to participate in research as White patients, there continue to be disparities in their representation in research [27-29]. This can be attributed to multilevel barriers to participation, which can range from individual (eg, distrust, lack of study awareness), interpersonal (eg, health professional biases), and systemic factors (eg, inequities in health care access and research inclusion) [26,29-32]. In our study, for example, the UCSF EHR produced a significantly lower proportion of eligible racial or ethnic minorities (even though our eligibility criteria were especially broad) to contact and invite into the Health eHeart Study.

Furthermore, patients with Medicaid or “unknown or declined to state” insurance were less likely to register compared to those with commercial insurance. However, there was a significant drop-off at the consent stage observed among patients with Medicaid insurance. Systemic factors such as lack of adequate insurance coverage and internet access are especially burdensome for racial or ethnic minorities and those with lower SES. Patients’ state of insurance coverage may be an indicator of their digital research participation [23,27-29,33]. In addition, the “digital divide” or disparity in internet access (ie, a growing share of Americans that are low-income, Black, or Latino are becoming smartphone-only internet users) also hinder the participation of underrepresented groups in digital health studies [34-36]. The recruitment of underrepresented groups in research will require higher-touch approaches with significant investment in relationship building to overcome persistent barriers.

Most medical conditions that we studied, and medical comorbidity burden in general, did not appear to be obstacles to enrollment after adjustment for other factors. Patients with a low comorbidity burden, either CCI score of 1 or 2, were more likely to register compared to those with no relevant comorbidities. This contrasts with the classic “healthy volunteer” effect that suggests those who join clinical trials or studies are healthier than those who decide not to participate [37,38]. Unsurprisingly, due to the heart health focus of the study, patients with congestive heart failure were more likely to register compared to those without the condition. Similarly, patients with dementia and several other conditions were less likely to enroll after adjustment for comorbidity burden. There is limited literature on the clinical makeup of participants who are remotely recruited to join digital health studies, much less the entire population reached by their remote recruitment efforts.

Operational considerations of a recruitment plan are also important for maximizing study enrollment [39]. Incorporating follow-up schemas in recruitment campaigns can help research teams boost their chances of reaching and enrolling people from a given contact list. Active patients are shown to be more receptive to email invitations from their current health care system. Narrowing recruitment to a presumably more engaged or readily accessible sample can help optimize enrollment yields and conserve limited study resources.

### Implications

The findings from the study reveal a more nuanced understanding of using email as a digital health study recruitment tool. While there is no definitive recruitment strategy that fully addresses diversity shortcomings in digital health studies, the email campaign has demonstrated effective recruitment of certain underrepresented groups (eg, older adults and women) in contrast to prior studies that included email [16,40]. The study also provides extensive participant characterization in contrast to other studies of digital recruitment tool effectiveness [15,41,42]. While our findings are most pertinent to email recruitment, other digital channels such as SMS text message, phone calls, or web-based chat may have differential (and underexplored) potential in recruiting diverse populations. Multichannel recruitment can help better reach underrepresented groups, but it also requires procuring additional sensitive information about the recruited population. Future email campaigns may need to carry out participant-centered research to help optimize trust and cocreate messaging, including in partnership with community organizations, patient advocacy groups, or charity organizations. To adapt recruitment strategies to be more inclusive and equitable, study teams need to understand the fundamental needs of target recruited populations (ie, those considered underrepresented or vulnerable populations in research, have rare disease conditions, and people from low- and middle-income countries) and address enrollment barriers (eg, lessen time and economic burden of study procedures and provide recruitment materials in various languages). Once email campaign procedures and messaging are deemed appropriate and inclusive, these partnering groups can also help further expand the reach of study recruitment efforts by engaging members from their respective email listservs.

Finally, recruitment is only the beginning of the digital research study life cycle. Retention and engagement of enrolled underrepresented participants throughout a study’s life course are equally important for generating equitable health discoveries and benefits. Digital research studies need to be inclusive by design and supported in ongoing initiatives to achieve equity at all stages of participation. For the next generation of “precision population health” eCohort studies, such as the NIH’s one-million-person *All of Us* Research Program [43], recruitment strategies will need to be as dynamic as the diverse populations they hope to reach to break through established barriers to digital health study participation.

### Limitations

This study had important limitations. First, the way in which patients were included in our recruitment campaign may be a source of selection bias. Despite Health eHeart’s broad eligibility criteria, UCSF patients with documented email addresses were also those with MyChart patient portal accounts, for which there are known baseline differences across patient populations. There are also inherent differences among patients who list English as their preferred language compared to those who do not. Second, recruitment was restricted to UCSF patients who are not representative of the regional or general population. It has also been reported that “university-led” studies have higher levels of participation [18]. Thus, enrollment disparity patterns observed in our analysis may vary greatly if the email recruitment campaign were deployed to the general population. Third, while the subject line messaging and delivery days and times were varied as part of the recruitment outreach strategy, the content of the email remained constant. For example, the image used in the email might have been varied with consideration of patient-physician concordance [44]. It is recommended that subsequent email initiatives will need to further tailor and fine-tune recruitment materials to better resonate with the recruited population. Fourth, our study did not consider the potential role of interactions in our analyses. Finally, although our email recruitment campaign occurred in 2015-2016 and digital research participation patterns may have changed since then, there remain limited comprehensive evaluations on the effectiveness of email for digital health study recruitment. This study offers important insights into the potential returns on participation when study teams invest resources in using email for recruitment. However, it remains to be seen whether the effect of email recruitment will be further diluted considering the COVID-19 pandemic [45-48] and amidst a digital landscape in excess of outlets vying for one’s attention.

### Conclusions

The purpose of this study was to understand who volunteers to participate in a digital health study because of an exclusive and large-scale remote email recruitment campaign. Contrary to other mass media campaign evaluations, our study was able to characterize the sample of patients who did and did not enroll, whereas previous evaluations had limited to no information about the latter group. Overall, the findings showed that enrollment was driven more strongly by sociodemographic than clinical factors. Email is also an extremely efficient means of recruiting participants from a large list into the Health eHeart Study. Despite some improvements in representation, the formulation of truly diverse studies will require additional resources and strategies to overcome remaining participation barriers.

## Appendix

Multimedia Appendix 1 Health eHeart Study recruitment email delivered to eligible UCSF patients.

Multimedia Appendix 2 Email campaign details for initial wave and follow-up wave.

Multimedia Appendix 3 Unadjusted odds of registration among contacted patients (left) and consent among registered patients (right).

## Abbreviations

aOR: adjusted odds ratio
API: Asian and Pacific Islander
CCI: Charlson Comorbidity Index
eCohort: electronic cohort
EHR: electronic health record
SES: socioeconomic status
UCSF: University of California, San Francisco

## Data Availability

Data sets generated and analyzed during this study are not publicly available due to the presence of sensitive information.
