# Supplementary figures and images for: Email-Based Recruitment Into the Health eHeart Study: Cohort Analysis of Invited Eligible Patients
Source: J Med Internet Res. 2023 Dec 22;25:e51238. doi: 10.2196/51238 (PMC10770794; doi:10.2196/51238)

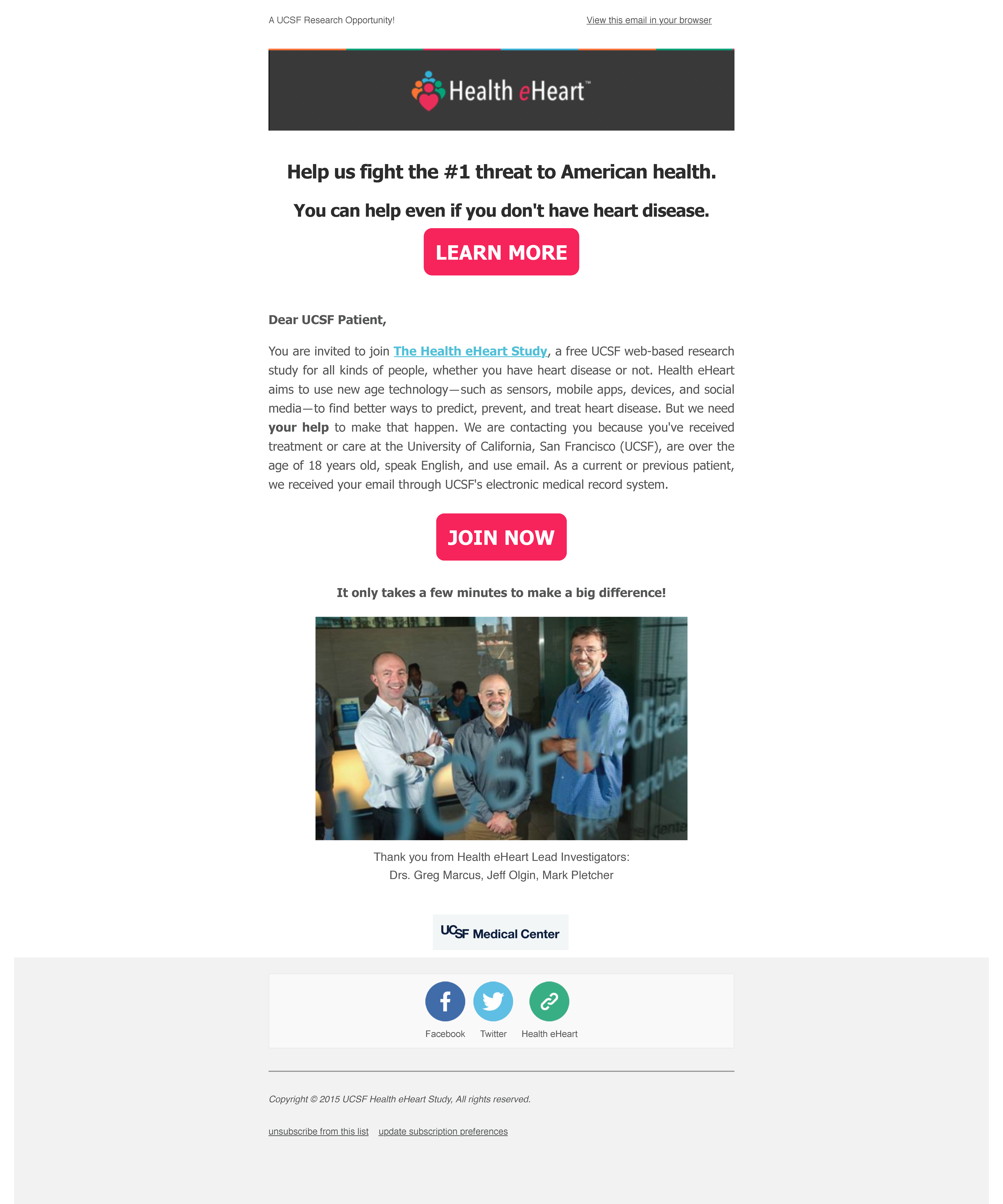

Supplement: Multimedia Appendix 1 [file jmir_v25i1e51238_app1.png]

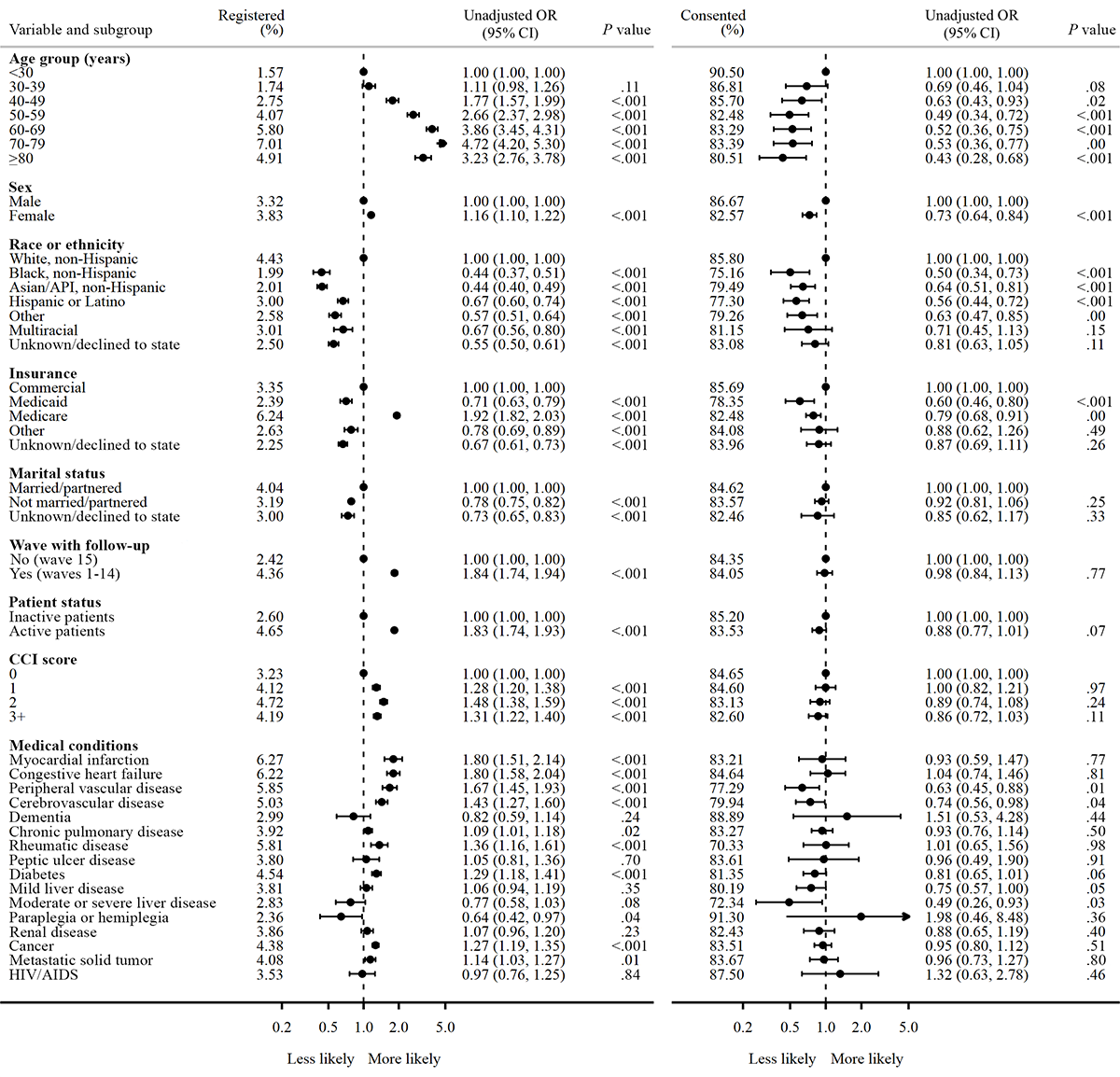

Supplement: Multimedia Appendix 3 [file jmir_v25i1e51238_app3.png]
