# Supplementary material for: Email-Based Recruitment Into the Health eHeart Study: Cohort Analysis of Invited Eligible Patients
Source: J Med Internet Res. 2023 Dec 22;25:e51238. doi: 10.2196/51238 (PMC10770794; doi:10.2196/51238)
Supplement: Multimedia Appendix 2 [file jmir_v25i1e51238_app2.docx]

**Multimedia Appendix 2.** Email campaign details for initial wave and follow-up wave

| **Initial** | **Subject Line** | **Day** | **Date** | **Time** | **Sent** | **Bounced** | **Delivered** | **Registered** | **Consented** |
| --- | --- | --- | --- | --- | --- | --- | --- | --- | --- |
| Wave 1 | Option 1 | Wed | 08/12/15 | 5:00 PM | 983 | 71 | 912 | 23 | 18 |
| Wave 2 | Option 1 | Wed | 08/19/15 | 1:30 PM | 990 | 53 | 937 | 23 | 19 |
| Wave 3 | Option 2 | Thu | 08/20/15 | 9:00 AM | 978 | 57 | 921 | 11 | 10 |
| Wave 4 | Option 1 | Fri | 08/21/15 | 9:00 AM | 989 | 67 | 922 | 26 | 24 |
| Wave 5 | Option 1 | Sat | 08/22/15 | 9:00 AM | 983 | 69 | 914 | 19 | 16 |
| Wave 6 | Option 1 | Sun | 08/23/15 | 9:00 AM | 986 | 56 | 930 | 14 | 12 |
| Wave 7 | Option 1 | Mon | 08/24/15 | 9:00 AM | 983 | 40 | 943 | 31 | 25 |
| Wave 8 | Option 1 | Tue | 08/25/15 | 9:00 AM | 980 | 55 | 925 | 26 | 22 |
| Wave 9 | Option 1 | Wed | 08/26/15 | 9:00 AM | 980 | 55 | 925 | 21 | 15 |
| Wave 10 | Option 1 | Thu | 08/27/15 | 9:00 AM | 984 | 53 | 931 | 24 | 20 |
| Wave 11 | Option 1 | Wed | 08/26/15 | 1:00 PM | 19,728 | 1,242 | 18,486 | 456 | 388 |
| Wave 12 | Option 3 | Fri | 09/04/15 | 10:00 AM | 29,563 | 1,885 | 27,678 | 791 | 675 |
| Wave 13 | Option 3 | Wed | 09/09/15 | 5:30 PM | 39,419 | 2,567 | 36,852 | 1,238 | 1,062 |
| Wave 14 | Option 4 | Wed | 09/30/15 | 8:00 AM | 29,573 | 1,771 | 27,802 | 622 | 528 |
| Wave 15 | Option 4 | Mon | 02/08/16 | 9:00 AM | 78,864 | 5,407 | 73,457 | 1,776 | 1,498 |
| **TOTAL** |  |  |  |  | **206,983** | **13,448** | **193,535** | **5,101** | **4,332** |
|  |  |  |  |  |  |  |  |  |  |
| **Follow-up** | **Subject Line** | **Day** | **Date** | **Time** | **Sent** | **Bounced** | **Delivered** | **Registered** | **Consented** |
| Wave 1 | Option 5 | Wed | 08/26/15 | 2:00 PM | 886 | 10 | 876 | 18 | 17 |
| Wave 2 | Option 5 | Wed | 08/26/15 | 2:00 PM | 918 | 11 | 907 | 14 | 13 |
| Wave 3 | Option 5 | Thu | 09/03/15 | 8:00 AM | 903 | 8 | 895 | 19 | 12 |
| Wave 4 | Option 5 | Fri | 09/04/15 | 8:00 AM | 898 | 16 | 882 | 12 | 12 |
| Wave 5 | Option 5 | Thu | 09/03/15 | 8:00 AM | 901 | 10 | 891 | 14 | 10 |
| Wave 6 | Option 5 | Fri | 09/04/15 | 7:00 AM | 913 | 9 | 904 | 11 | 10 |
| Wave 7 | Option 5 | Fri | 09/04/15 | 8:00 AM | 913 | 8 | 905 | 14 | 9 |
| Wave 8 | Option 5 | Fri | 09/04/15 | 9:00 AM | 902 | 12 | 890 | 12 | 11 |
| Wave 9 | Option 5 | Fri | 09/04/15 | 10:00 AM | 897 | 6 | 891 | 14 | 12 |
| Wave 10 | Option 5 | Fri | 09/04/15 | 11:00 AM | 905 | 8 | 897 | 12 | 8 |
| Wave 11 | Option 5 | Wed | 09/02/15 | 4:00 PM | 18,054 | 233 | 17,821 | 396 | 314 |
| Wave 12 | Option 4 | Fri | 10/02/15 | 8:00 AM | 26,995 | 408 | 26,587 | 393 | 342 |
| Wave 13 | Option 4 | Wed | 09/30/15 | 4:00 PM | 35,695 | 490 | 35,205 | 482 | 397 |
| Wave 14 | Option 5 | Mon | 10/19/15 | 11:00 AM | 27,185 | 325 | 26,860 | 500 | 400 |
| Wave 15 | - | - | - | - | - | - | - | - | - |
| **TOTAL** |  |  |  |  | **116,965** | **1,554** | **115,411** | **1,911** | **1,567** |

*Option 1: A UCSF Research Opportunity to Fight Heart Disease!*

*Option 2: Join UCSF in the fight against heart disease!*

*Option 3: UCSF's Health eHeart Study Needs Your Help!*

*Option 4: Join UCSF's Health eHeart Study to Help Fight Heart Disease!*

*Option 5: UCSF's Health eHeart Study Still Needs Your Help!*
